# Painful subacute thyroiditis occurring after the administration of influenza vaccine and hyaluronic acid dermal filler: A case report

**DOI:** 10.1097/MD.0000000000029120

**Published:** 2022-03-18

**Authors:** Nailya R. Bulatova, Ayman A. Zayed, Ula Qasem Hijjawi, Satani G. Sharkas, Faris G. Bakri

**Affiliations:** ^a^ *Department of Biopharmaceutics and Clinical Pharmacy, School of Pharmacy, The University of Jordan, Amman, Jordan,* ^b^ *Department of Medicine, School of Medicine, Jordan University Hospital, Amman, Jordan,* ^c^ *Infectious Disease and Vaccine Center, The University of Jordan, Amman, Jordan.*

**Keywords:** case report, *HLA B-35* haplotype, hyaluronic acid, influenza vaccine, subacute thyroiditis

## Abstract

**Rationale:**

The occurrence of subacute thyroiditis (SAT) after vaccines or after hyaluronic acid skin fillers is very rare and might be related to genetic susceptibility. We suggest that the co-administration of both products could potentially increase the possibility of development of SAT.

**Patient concerns:**

A 58-year-old Caucasian healthy female initially presented with chills, myalgia, dysphagia, sore throat, dry cough, fatigue, and intermittent fever of 38.5°C orally after simultaneous injection of an influenza vaccine and a dermal filler containing hyaluronic acid. Ten days later the patient developed palpitations and neck pain radiating to the left jaw.

**Diagnosis and interventions:**

She was diagnosed with SAT on day 16 after her first visit and responded promptly to etoricoxib treatment.

**Outcomes:**

The patient progressed clinically from hyperthyroidism to euthyroid state and eventually to hypothyroidism and further testing showed she had *HLA B-35* haplotype.

**Lessons:**

Physicians should be aware that SAT might be associated with the administration an influenza vaccine and this possible association might increase if the vaccine was co-administered with a dermal filler.

## 1. Introduction

Painful subacute thyroiditis (SAT) is a self-limited inflammatory disorder. SAT begins with a prodrome of generalized myalgias, pharyngitis, low-grade fever, and fatigue. Patients then develop fever and severe neck pain, swelling, or both. Up to 50% of patients have symptoms of thyrotoxicosis. In most patients, thyroid function will be normal after several weeks of thyrotoxicosis, and hypothyroidism will subsequently develop, lasting 4 to 6 months. Although thyroid function normalizes spontaneously in 95% of patients over a period of 6 to 12 months, residual hypothyroidism persists in 5% of patients.^[1]^

SAT is presumed to be caused by a viral infection. The evidence for this comes from a tendency for the disease to follow upper respiratory tract infections or sore throats and from reports of disease clusters during outbreaks of viral infection and from studies of viral antibody titers. Interestingly, many patients were shown to have *HLA-B35* and because of this, it appears that the onset of SAT is genetically influenced and that SAT might occur through a susceptibility to viral infection in genetically predisposed individuals.^[2]^ The types of viruses associated with SAT include Coxsackie viruses, Echo viruses, adenoviruses, influenza viruses, mumps and rubella viruses, parvovirus B19, orthomyxovirus, human immunodeficiency virus, Epstein-Barr virus, hepatitis E, measles virus, dengue virus, and severe acute respiratory syndrome-coronavirus-2.^[3]^ Tissue examination shows that the follicles are often infiltrated, resulting in disrupted basement membrane and rupture of the follicles. The thyroid injury is thought to be the result of cytolytic T-cell recognition of viral and cell antigens present in an appropriate complex.^[2]^ In addition to the above viral causes, a few case reports showed that SAT could also be caused by tumor necrosis factor-a inhibitors adalimumab, etanercept, and infliximab.^[3]^

Other rare and possible causes for SAT include vaccines and biomaterials. Vaccines can cause autoimmune complications through several mechanisms including: postvaccination phenomena of autoimmune/inflammatory syndrome induced by adjuvants where the adjuvant, in a fraction of genetically susceptible and predisposed subjects, may lead to serious side-effects due to the activation of autoimmune pathways^[4]^; molecular mimicry of the infecting agent as when a structural similarity exists between some viral antigen (or other component of the vaccine) and a selfantigen^[5]^; increase in immune complexes^[5]^; exposure to other vaccine components such as neomycin and polymyxin^[5]^; and bystander activation which is an antigen nonspecific mechanism that leads to the activation of autoreactive T cells.^[6]^ Biomaterial injections and prostheses such as silicone and hyaluronic acid (HA) can trigger autoimmune complications by acting as a T celldirected antigen, as a superantigen, or as an adjuvant through autoimmune/inflammatory syndrome induced by adjuvants effect.^[7]^

## 2. Case presentation

A 58-year-old Caucasian healthy female, presented to the Hospital in November 2019 with a 3-day history of chills, myalgia, dysphagia, sore throat, dry cough, fatigue, and intermittent fever of 38.5°C orally for which she took acetaminophen. Four days before the onset of symptoms, the patient received an injection of Juviderm (HA) dermal filler into the nasolabial area of the face. One day after the filler injection, she received an injection of seasonal influenza vaccine [Influvac 2019/2020; batch number T05, Abbott, containing the following strains: A/Brisbane/02/2018/(H1N1)pdm2009-like strain; A/Kansas/14/2017/(H3N2)-like strain; B/Colorado/06/2017-like strain]. She was feeling well during the time of vaccination and dermal filler injection. The patient had received the influenza vaccine annually for more than ten years without any complication and she also received dermal fillers containing HA, namely Juviderm and Restylane, several times without any adverse events. On further inquiry, the patient denied any familial history of autoimmune diseases.

On physical examination, her blood pressure was 120/80mm Hg, pulse rate 84 beats per minute, and temperature 37.1°C orally. Her throat was congested. The rest of her physical examination was unremarkable. She had elevation of both Creactive protein (CRP) (50.6 mg/L; normal < 5mg/L) and erythrocyte sedimentation rate (ESR) (45mm/1st hour; normal < 20mm/1st hour). A chest radiograph was normal. The patient was subsequently advised to continue acetaminophen.

One week after presentation to the hospital, the patient developed anterior and right-sided neck swelling and tenderness with pain radiating to the right ear. The body temperature was 38.0°C orally. Laryngoscopy showed laryngeal erythema. Therefore, ciprofloxacin was prescribed at a dose of 500 mg bid for 10 days.

At the follow-up, symptoms did not improve, the patient started to have palpitations and neck pain radiating to the left jaw. A repeat laryngoscopy showed no additional findings. Dental examination and panorama were normal. An echocardiogram was normal. An electrocardiogram showed sinus tachycardia at 110 beats per minute for which the patient was prescribed bisoprolol in the dose of 5 mg qd.

Blood and urine cultures were negative. Blood film showed thrombocytosis and was negative for malaria parasite. Serology testing was negative for human immunodeficiency virus, hepatitis B, and C viruses, rickettsia, brucella, toxoplasma, rheumatoid factor, and antinuclear antibodies.

At day 19 of the symptom onset and day 16 of the initial presentation to our institution, a thyroid examination showed a tender small goiter. Serum thyroid stimulating hormone showed decreased level (0.321 mIU/L; normal 0.55-4.78 mIU/L) while serum free thyroxine and free triiodothyronine showed levels in the upper limit of normal range, (20.12 pmol/L; normal 11.5-22.7 pmol/L) and (6.35 pmol/L; normal 3.5-6.5 pmol/L), respectively. Antithyroglobulin antibody and thyroid peroxidase antibody were both increased at 125.6IU/mL (normal; < 4.11 (IU/mL) and 139.6IU/mL (normal; < 5.61 IU/mL), respectively. Ultrasound of the thyroid gland showed an enlarged heterogeneous thyroid gland with nodular background and increased vascularity suggestive of thyroiditis. Repeated ESR and CRP showed an increase to 100mm/1st hour and 75mg/L, respectively. Subsequently, the patient was diagnosed with painful SAT and was prescribed etoricoxib 90 mg qd. All symptoms dramatically resolved after 3 days. Human leukocyte antigen (HLA) typing performed by cytotoxic cross match showed *HLA-B35* haplotype.

Three weeks after the etoricoxib therapy, her CRP and ESR became normal and the patient became euthyroid. Two months after starting the therapy, the patient developed primary hypothyroidism. After 25 months from starting therapy, the patient is doing well and is being maintained on levothyroxine at a dose of 50 mcg qd with regular follow-up of her thyroid function tests.

## 3. Discussion

The development of SAT after the administration of either an influenza vaccine or a filler containing HA is very rare.^[7-15]^ To our knowledge, this is the first report of painful SAT that has occurred after the co-administration of an influenza vaccine and a dermal filler containing HA. Our patient experienced a rare presentation of SAT. The patient was diagnosed with SAT after a relatively long course of fever of 19 days. Indeed, SAT as a cause of fever can be missed and in some reports, SAT was shown to present as fever of unknown origin.^[16]^ The patient also progressed to develop permanent hypothyroidism which is uncommon in SAT.^[1]^ In addition, the patient showed genetic susceptibility as she carried *HLA-B35* haplotype. In about 70% of patients, SAT was associated with the *HLA-B-35* allele.^[17]^ Among the 8 patients who were reported to develop SAT after an influenza vaccine, only 3 underwent HLA testing and all the 3 patients showed *HLA-B35* haplotype including one who was reported from our institution.^[10-12]^ The influenza vaccine that was administered here was of a subunit, inactivated, trivalent nonadjuvanted type.^[18]^ Hence, we suggest that the viral subunits were able to activate the immune system and cause SAT. In fact, previous studies had pointed to the etiologic role of influenza viruses in thyroiditis.^[3]^

Reported cases of thyroiditis after vaccines are scarce, so far, a total of 53 cases of postvaccination thyroiditis were described; 41 cases after human papillomavirus vaccine, 8 cases after influenza vaccine,^[8-15]^ 1 case after hepatitis B virus vaccine,^[4]^ and 3 cases after severe acute respiratory syndrome-coronavirus-2 vaccine.^[19,20]^

The incidence of adverse effects for HA is rare and is estimated to be between 0.3% and 0.4%. Early onset inflammatory adverse events start within 2 to 4 weeks after injection. Late onset inflammatory adverse events appear between 1 and 60 months postimplantation with 14 months as mean period. Most of the late onset adverse events present as an inflammatory response.^[21]^ Immune mediated adverse effects related to HA dermal fillers are rare and include inflammatory nodules, cutaneous vasculitis, panniculitis, thyroiditis, scleromyxedema, and sarcoid like reaction.^[7,22]^ In some cases, these late reactions had resulted from a second triggering effect of other adjuvants, particularly other fillers, clinical or subclinical infections, local trauma, or vaccines.^[7]^ Recently, few reports have described the occurrence of cutaneous reactions after coronavirus disease-19 vaccination in patients injected with dermal fillers.^[23-27]^

Autoimmune thyroiditis after injection of skin fillers due to HA is very rare. Alijotas-Rerig et al^[7]^ examined a Spanish cohort of 45 cases of late-onset inflammatory adverse reactions related to fillers/implant defined as 3 months or longer after treatment. The researchers found autoimmune thyroiditis in 13/45 patients; in 2 of those cases, the constituent was HA.

Here, the onset of SAT symptoms occurred after 3 days of the administration of an influenza vaccine and an HA containing dermal filler. Because the inflammatory thyroid complication of the influenza vaccines occur within a range of 2 days to 8 weeks of injection^[8-15]^ and those of HA occur after several months of injection,^[7]^ SAT in this case probably had resulted either from the influenza vaccine alone or from the influenza vaccine and the HA as a second triggering factor. We favor the latter idea that HA was indeed a second triggering factor because all previously administered influenza vaccines in this patient did not cause complications and because the occurrence of SAT after vaccines is a rare event and so the exposure to another factor such as HA would be suspected to play a role in increasing this rare possibility.

Sayre et al^[28]^ noted that although case reports do not rank superior among different study designs, as they frequently describe a single patient, they might carry substantial educational value and advance medical knowledge. Likewise, our case report is designed to show unusual features identified in clinical settings and to potentially generate new research questions.

## 4. Conclusions

Physicians should be aware that SAT might be associated with the administration an influenza vaccines and this possible association might increase if the vaccine was co-administered with dermal fillers. Further research is needed to explore the possible association of influenza vaccine with SAT.

## Author contributions

**Conceptualization:** Nailya R Bulatova, Ayman A Zayed, Faris G Bakri.

**Data curation:** Nailya R Bulatova, Ula Qasem Hijjawi, Satani G Sharkas, Faris G Bakri.

**Methodology:** Nailya R Bulatova, Ayman A Zayed.

**Writing** - **original draft:** Nailya R Bulatova, Ayman A Zayed, Ula Qasem Hijjawi, Satani G Sharkas, Faris G Bakri.

**Writing** - **review & editing:** Nailya R Bulatova, Faris G Bakri.

## References

[1] Pearce E Farwell A Braverman LE. Thyroiditis. N Engl J Med 2003;348:2646-55. 12826640 10.1056/NEJMra021194

[2] Desailloud R Hober D. Viruses and thyroiditis: an update. Virol J 2009;6:5. 19138419 10.1186/1743-422X-6-5 PMC2654877

[3] Stasiak M Lewinski A. New aspects in the pathogenesis and management of subacute thyroiditis. Rev Endocr Metab Disord 2021; 22:1027-39. 33950404 10.1007/s11154-021-09648-y PMC8096888

[4] Bragazzi N Hejly A Watad A Adawi M Amital H Shoenfeld Y. ASIA syndrome and endocrine autoimmune disorders. Best Pract Res Clin Endocrinol Metab 2020;34:101412. 32265102 10.1016/j.beem.2020.101412

[5] Watad A David P Brown S Shoenfeld Y. Autoimmune/inflammatory syndrome induced by adjuvants and thyroid autoimmunity. Front Endocrinol (Lausanne) 2017;7:150. 28167927 10.3389/fendo.2016.00150 PMC5256113

[6] Olivieri B Betterle C Zanoni G. Vaccinations and autoimmune diseases. Vaccines 2021;9:815. 34451940 10.3390/vaccines9080815 PMC8402446

[7] Alijotas-Reig J Esteve-Valverde E Gil-Aliberas N Garcia-Gimenez V. Autoimmune/inflammatory syndrome induced by adjuvants-ASIArelated to biomaterials: analysis of 45 cases and comprehensive review of the literature. Immunol Res 2018;66:120-40. 29199390 10.1007/s12026-017-8980-5

[8] Hsiao J Hsin S Hsieh M Hsia P Shin S. Subacute thyroiditis following influenza vaccine (Vaxigrip) in a young female. Kaohsiung J Med Sci 2006;22:297-300. 16793568 10.1016/S1607-551X(09)70315-8 PMC11917749

[9] Girgis C Russo RR Benson K. Subacute thyroiditis following the H1N1 vaccine. J Endocrinol Invest 2010;33:506. 20671410 10.1007/BF03346633

[10] Martinez J Corder E Uzcategui M Garcia M Sostre S Garcia A. Subacute thyroiditis and dyserythropoesis after influenza vaccination suggesting immune dysregulation. Bol Assoc Med PR 2011;103: 48-52. 22111471

[11] Yakushiji F. Subacute thyroiditis after seasonal influenza vaccination. Drugs Ther Stud 2011;1:33-4.

[12] Momani M Zayed A Bakri F. Subacute thyroiditis following influenza vaccine: a case report and literature review. Ital J Med 2015;9:384-6.

[13] Altay F Güz G Altay M. Subacute thyroiditis following seasonal influenza vaccination. Hum Vaccin Immunother 2016;12:1033-4. 26809709 10.1080/21645515.2015.1117716 PMC4962945

[14] Guzmán-García S Domínguez-Moreno R Rojas-De Ita I, et al. Subacute thyroiditis following influenza vaccine. Rev Mex Endocrinol Metab Nutr 2016;3:34-8.

[15] Passah A Arora S Damle N Reddy K Khandelwal D Aggarwal S. Occurrence of subacute thyroiditis following influenza vaccination. Indian J Endocrinol Metab 2018;22:713-4. 30294587 10.4103/ijem.IJEM_237_18 PMC6166570

[16] Al-Tikrity M Magdi M Abou Samra A Elzouki A. Subacute thyroiditis: an unusual presentation of fever of unknown origin following upper respiratory tract infection. Am J Case Rep 2020;21: e920515. 32127513 10.12659/AJCR.920515 PMC7069329

[17] Stasiak M Tymoniuk B Michalak R Stasiak B Kowalski M Lewiński A. Subacute thyroiditis is associated with *HLA-B***18:01, - DRB1***01 and - C***04:01* - the significance of the new molecular background. J Clin Med 2020;9:534. 32079059 10.3390/jcm9020534 PMC7074389

[18] Tregoning J Russell R Kinnear E. Adjuvanted influenza vaccines. Hum Vaccin Immunother 2018;14:550-64. 29232151 10.1080/21645515.2017.1415684 PMC5861793

[19] Franquemont S Galvez J. Subacute thyroiditis after mRNA vaccine for COVID-19. J Endocr Soc 2021;5(Suppl 1):A956-7.

[20] Vera-Lastra O Navarro A Cruz Domiguez M Medina G Sanchez Valadez T Jara L. Two cases of Graves’ disease following SARS-CoV-2 vaccination: an autoimmune/inflammatory syndrome induced by adjuvants. Thyroid 2021;31:1436-9. 33858208 10.1089/thy.2021.0142

[21] Decates T Velthuis P Schelke L, et al. Increased risk of late-onset, immune-mediated, adverse reactions related to dermal fillers in patients bearing *HLA-B* **08 and DRB1* **03* haplotypes. Dermatol Ther 2020;34: e14644. 33300274 10.1111/dth.14644

[22] Alijotas-Reig J Garcia-Gimenez V. Delayed immune-mediated adverse effects related to hyaluronic acid and acrylic hydrogel dermal fillers: clinical findings, long-term follow-up and review of the literature. J Eur Acad Dermatol Venereol 2008;22:150-61. 18211407 10.1111/j.1468-3083.2007.02354.x

[23] Savva D Battineni G Amenta F Nittari G. Hypersensitivity reaction to hyaluronic acid dermal filler after the Pfizer vaccination against SARS-CoV-2. Int J Infect Dis 2021;113:233-5. 34597761 10.1016/j.ijid.2021.09.066 PMC8479353

[24] Michon A. Hyaluronic acid soft tissue filler delayed inflammatory reaction following COVID-19 vaccination - a case report. J Cosmet Dermatol 2021;20:2684-90. 34174156 10.1111/jocd.14312 PMC8447415

[25] Osmond A Kenny B. Reaction to dermal filler following COVID-19 vaccination. J Cosmet Dermatol 2021;20:3751-2. 34706150 10.1111/jocd.14566 PMC8661616

[26] Rauso R Lo Giudice G Zerbinati N Nicoletti G Fragola R Tartaro G. Adverse events following COVID-19 vaccine in patients previously injected with facial filler: scoping review and case report. Appl Sci 2021;11:10888.

[27] Ghasemi S Dashti M. Fight against COVID-19 with mRNA vaccines and interaction with Dermal fillers. Clin Exp Vaccine Res 2021;10: 151-3. 34222128 10.7774/cevr.2021.10.2.151 PMC8217583

[28] Sayre JW Toklu HZ Ye F Mazza J Yale S. Case reports, case series - from clinical practice to evidence-based medicine in graduate medical education. Cureus 2017;9:e1546. 29018643 10.7759/cureus.1546 PMC5630458

